# Sarcopenia as a predictor of mortality among the critically ill in an intensive care unit: a systematic review and meta-analysis

**DOI:** 10.1186/s12877-021-02276-w

**Published:** 2021-06-02

**Authors:** Xiao-Ming Zhang, Denghong Chen, Xiao-Hua Xie, Jun-E Zhang, Yingchun Zeng, Andy SK Cheng

**Affiliations:** 1 Department of Nursing, Chinese Academy of Medical Sciences - Peking Union Medical College, Peking Union Medical College Hospital (Dongdan campus), 100730 Beijing, China; 2 The Third Affiliated Hospital of Guangdong Medical University (LongJiang hospital of Shunde District, Foshan, Guangdong China; 3 Department of Nursing, The Second People’s Hospital of Shenzhen, The First Affiliated Hospital of Shenzhen University, Shenzhen, China; 4 School of Nursing, Sun Yat-sen University, Guangzhou, China; 5 Department of Nursing, The Third Affiliated Hospital of Guangzhou Medical University, Guangzhou, China; 6 Department of Rehabilitation Sciences, The Hong Kong Polytechnic University, Hong Kong, China

**Keywords:** Sarcopenia, Mortality, Critically ill, Intensive care unit, Meta-analysis

## Abstract

**Background:**

The evidence of sarcopenia based on CT-scan as an important prognostic factor for critically ill patients has not seen consistent results. To determine the impact of sarcopenia on mortality in critically ill patients, we performed a systematic review and meta-analysis to quantify the association between sarcopenia and mortality.

**Methods:**

We searched studies from the literature of PubMed, EMBASE, and Cochrane Library from database inception to June 15, 2020. All observational studies exploring the relationship between sarcopenia based on CT-scan and mortality in critically ill patients were included. The search and data analysis were independently conducted by two investigators. A meta-analysis was performed using STATA Version 14.0 software using a fixed-effects model.

**Results:**

Fourteen studies with a total of 3,249 participants were included in our meta-analysis. The pooled prevalence of sarcopenia among critically ill patients was 41 % (95 % CI:33-49 %). Critically ill patients with sarcopenia in the intensive care unit have an increased risk of mortality compared to critically ill patients without sarcopenia (OR = 2.28, 95 %CI: 1.83–2.83; P < 0.001; I^2^ = 22.1 %). In addition, a subgroup analysis found that sarcopenia was associated with high risk of mortality when defining sarcopenia by total psoas muscle area (TPA, OR = 3.12,95 %CI:1.71–5.70), skeletal muscle index (SMI, OR = 2.16,95 %CI:1.60–2.90), skeletal muscle area (SMA, OR = 2.29, 95 %CI:1.37–3.83), and masseter muscle(OR = 2.08, 95 %CI:1.15–3.77). Furthermore, critically ill patients with sarcopenia have an increased risk of mortality regardless of mortality types such as in-hospital mortality (OR = 1.99, 95 %CI:1.45–2.73), 30-day mortality(OR = 2.08, 95 %CI:1.36–3.19), and 1-year mortality (OR = 3.23, 95 %CI:2.08 -5.00).

**Conclusions:**

Sarcopenia increases the risk of mortality in critical illness. Identifying the risk factors of sarcopenia should be routine in clinical assessments and offering corresponding interventions may help medical staff achieve good patient outcomes in ICU departments.

**Supplementary Information:**

The online version contains supplementary material available at 10.1186/s12877-021-02276-w.

## Background

Critically ill patients in intensive care units often suffered from multiple organ dysfunction, which increased the risk of mortality. Mortality rates - especially among oncology and hematology patients - have steadily decreased over time, thanks to dramatic progress in medical care [1]. However, the mortality of critically ill patients is still one of the most important issues, especially for the elderly patients with comorbidities and functional decline. There are several reasons that account for critically ill patients’ mortality, such as malnutrition [2], sepsis [3], immobility, and multiple organ dysfunction syndrome [4]. Therefore, it is important to predict mortality and stratify the risk of death. For decades, numerous scoring systems have been developed to predict clinical outcomes in critical illness, such as Sequential Organ Failure Assessment (SOFA) [5], systemic inflammatory response syndrome [6], and Acute Physiology and Chronic Health Evaluation APACHE-II [7]. However, these scoring systems have shown relatively poor predictable performance. Therefore, further studies are required to investigate more precise parameters in order to better predict poor clinical outcomes.

Sarcopenia is characterised by declining loss of muscle mass, strength, and physical function [8]. There is an increasing number of studies that show critically ill patients usually suffer from sarcopenia, due to factors such as nutritional status, inflammation, coexistence of disease, and inactivity [9]. It is estimated that the prevalence of sarcopenia is approximately 30-70 % in intensive care units [10, 11]. Sarcopenia has been confirmed to have an association with adverse clinical outcomes, such as falls, fractures, poor quality of life, mortality, and cognitive dysfunction among older adults in the community, nursing homes, or ICU [12–15]. Recently, Xia and colleagues published a meta-analysis concluding that injured patients with sarcopenia are at increased risk of mortality, with a two-fold increased risk compared to patient groups without sarcopenia [16]. Only four studies were conducted in ICU departments. Additionally, a number of recent studies have explored the relationship between sarcopenia and mortality in critically ill patients in the ICU. Some have shown that sarcopenia significantly increases mortality risk [10, 17–21], while others found no such an association [11, 22–25]. Given the inconsistent results, it is necessary to synthesize the evidence to explore the role sarcopenia may play in mortality in critically ill patients in the ICU. Therefore, we conducted a systematic review and meta-analysis to confirm whether critically ill patients with sarcopenia are at increased risk of mortality in ICU departments, which could inform prognostication on critically ill patients.

## Methods

This study was conducted and reported according to the Preferred Reporting Items for Systematic Reviews (PRISMA) guidelines [26], with a detailed checklist shown in supplementary Table 1. We have registered our protocol in PROSPERO with the number of CRD42020211548. The GRADE (Grading of Recommendations, Assessment, Development and Evaluation) approach was used to categorize the level of evidence.

### Search strategy

Two authors (XMZ, DHC) independently searched the electronic database, including PubMed, Embase, and Cochrane CENTRAL Library, from database inception until June 15, 2020. The search strategy includes keywords and medical subject headings (Mesh), including sarcopenia, critically ill (critical illness), or intensive care. The detailed search methods are shown in Supplemental File 1. We conducted a manual search of additional references from the selected articles. In addition, we searched the grey studies from Google and contacted with the authors of the unpublished study. The search results were discussed and confirmed by our team.

### Study selection

All of the articles identified from the electronic database were independently evaluated by two authors (YCZ and DHC). To start with, title and abstract were screened to confirm whether an article was potentially relevant. After that, the two authors checked the full text to identify whether it met the priority eligibility criteria. Any disagreements were addressed through team discussion and consensus.

### Inclusion and exclusion criteria

We established a priori inclusion criteria as follows:(i)The study design included observational studies; (ii) We included participants 18 years of age and over from ICU departments; (iii) The study includes a clear definition of sarcopenia using a consensual method or the prevalence of sarcopenia; and (iv) The study has shown the relationship between sarcopenia and mortality (30-day mortality, in-hospital mortality, 1-year mortality).

The exclusion criteria are as follows: (i) Article type: Conference, review articles, letters and laboratory research, case report; (ii) Using the sarcopenia index calculated as [(serum creatinine/serum cystatin C)x100] to report the association between SI (sarcopenia and index) and mortality; (iii) LMM (low muscle mass) was used as a continuous variable to report the association, but did not provide the results of association, between sarcopenia and mortality; (iv) Duplicate publication of articles; and (v) Languages other than English.

### Data extraction

The two authors (ASKC and XHX) independently used Microsoft® Excel 2016 to collect all data. The variables of the studies that were included were extracted as follows: Country, year of publication, time of CT scan, male/female ratio, age, prevalence of sarcopenia, sample size, definition of sarcopenia, cause of admission, study design and outcome. If the study reported more than two terms of mortality, such as 30-day mortality, 1-year mortality, the latter term was included. If mortality was shown in a multivariate analysis, we adopted the adjusted model, otherwise we calculated it. All data were checked by the two authors to achieve the final results.

### Quality assessment and risk of bias

Study quality assessment was evaluated by both authors using the Newcastle-Ottawa Scale (NOS). It included six aspects, with the scale’s total score, nine points [27]. The following NOS information included (i) Representativeness of the exposed cohort, (ii) comparability of the group, (iii) blinding of the investigators who measured the outcomes, (iv) time and completeness of follow-up, (v) contamination bias, and (vi) other potential sources of bias.

### Statistical analysis

Both authors (XMZ and ASKC) independently used STATA Version 14.0 software to calculate pooled data and heterogeneity. The studies that were included reported Odds ratio (OR), and 95 % confidence intervals (95 % CI) were extracted for future meta-analysis. We converted the effect of OR to ln(OR) for ratios in a meta-analysis, and subgroup analyses were conducted on different types of participants and outcomes. We performed Cochran’s Q test to examine statistical heterogeneity by using chi-square and I^2^ statistics. The I² statistic describes the percentage of variation across studies due to heterogeneity. Low, moderate, and high heterogeneity are defined by 25 %, 50 %, and 75 % cut-off (I^2^ values) respectively. If the I^2^ > 50 % or *p* < 0.10, we defined these studies as having significant heterogeneity and used the random-effects model. Otherwise, the fixed-effects model was used. Furthermore, Begg’s and Egger’s tests were used to identify whether any publication bias existed, and sensitivity analysis was conducted to assess the stability of the results. We also used trial sequential analysis (TSA) to assess whether the results were robust and reliable [28].

### Assessment of evidence quality

We displayed the evidence for each outcome using the methods recommended by GRADE. We rated the overall quality of evidence according to four categories: “high”, “moderate”, “low or very low”. These criteria were based on the evaluation of identified risks of bias, indirectness, imprecision, inconsistency, and publication bias.

## Results

### Study selection

Our team initially searched 594 articles from three Internet databases. After removing the duplicates, 497 articles remained. YCZ and DHC then screened the titles and abstracts, deleting 469 irrelevant studies. A further 28 articles were screened for full-text assessment: six studies were reviews or case studies and four studies were conference papers. In addition, four studies were excluded for not reporting the association between sarcopenia and mortality, as they only provided the association between skeletal muscle index (SMI) as a continuous variable and mortality [29–32]. Therefore, 14 publications were finalised for analysis. (Shown in Figure S1).

### Study summary

**Fig. 1:**
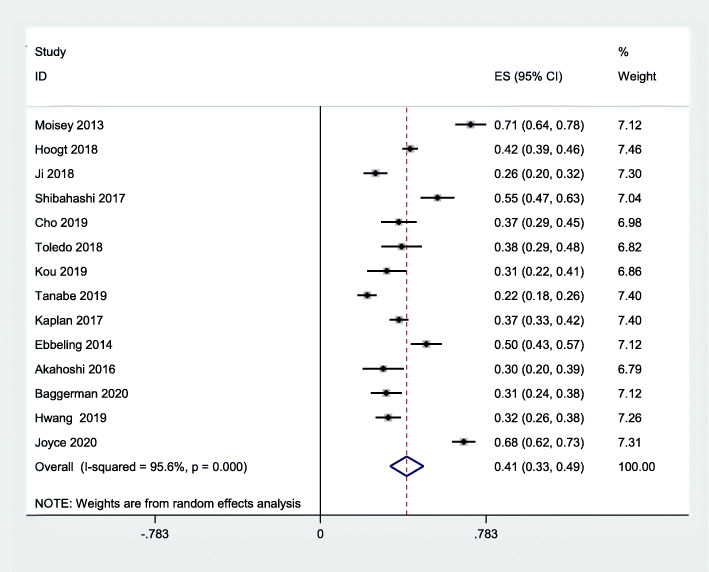
Pooled prevalence of sarcopenia in critically ill patients

There were 14 studies with a total of 3,249 patients included in our meta-analysis. All of the studies that were included were retrospective cohort studies, with the exception of one [33], which was a prospective cohort study. A total of five studies was conducted in the US [10, 22, 25, 33, 34]; while China [21, 35], Japan [17, 24], and the Netherlands [11, 18] each had two studies, and Korea [20], Australia [23], and Brazil [19] each had one study respectively. All studies used CT. to detect sarcopenia. There were several outcomes reported in our meta-analysis. Seven studies used in-hospital mortality [10, 11, 17, 18, 21, 22, 25], four studies used 30-day mortality [19, 23, 24, 35], and three studies used 1-year mortality [20, 33, 34]. The proportion of males among the studies that were included ranged from 51.40 to 69% (Table 1). Table S2 showed the result of each study with adjusted covariates. The pooled prevalence of sarcopenia among critically ill patients was 41 % (95 % CI: 33 -49 %; *p* = 0.000; I^2^ = 95.6 %) (Fig. 1).

**Table 1:** Summary of Included Studies on sarcopenia Associated with Mortality among critically ill

Author/year	Time of CT scan	Country	Design	Study interval	Male	Cause of ICU	Prevalence	Samplesize	Age/years	Sarcopenia Criteria	Outcome assessed	Method
Moisey 2013 [10]	On the day of admission	USA	retrospective cohort study	2009–2010	57 %	Trauma	71.00 %	149	79 (72–85)	SMI	in-hospital mortality	CT
Hoogt 2018[18]	NA	Netherlands	retrospective cohort study	2013–2014	53.40 %	Critically ill	42.4 %.	687	None	SMI	in-hospital mortality	CT
Ji 2018 [35]	Before ICU admission	China	retrospective cohort study	2012–2016	58.80 %	Sepsis	26.00 %	236	≥ 18	SMI	30-day survival	CT
Shibahashi 2017 [17]	On the day of admission	Japan	retrospective cohort study	2012–2016	69 %	Sepsis	55.3 %	150	75 (68, 82)	SMA	in-hospital mortality	CT
Cho 2019 [20]	1 day before admission	Korea	retrospective cohort study	2014–2017	61.40 %	Critically ill	37.00 %	127	≥18	TPA	1 year mortality	CT
Toledo 2018 [19]	Within 72 h or more after ICU admission	Brazil	retrospective cohort study	2010–2014	56 %	Critically ill	38.30 %	99	61.6(13.5)	SMI	30-day survival	CT
Kou 2019 [21]	Within 30 days of the first SBT	China	retrospective cohort study	2013–2014	56 %	Critically ill	31.30 %	96	73.0(63.0-80.8)	TPA	in-hospital mortality	CT
Tanabe 2019 [33]	Within 7 days of admission	USA	retrospective cohort study	2011–2014	51.40 %	Trauma	22.00 %	327	77.8 (8.6)	MCSA	1 year mortality	CT
Kaplan 2017 [34]	Within 2 days of admission	USA	retrospective cohort study	2011–2014	59.80 %	Trauma	37.10 %	450	≥ 65 years	SMI	1 year mortality	CT
Ebbeling 2014 [25]	On the day of admission	USA	Prespective cohort study	2005–2010	57 %	Trauma	50.00 %	180	≥ 55 years	TPA	in-hospital mortality	CT
Akahoshi 2016 [24]	Before ICU admission	Japan	retrospective cohort study	2012–2015	55.90 %	Trauma	29.70 %	84	None	SMA	30-day mortality	CT
Baggerman 2020 [11]	CT scan between − 6 and + 2 days from ICU admission	Netherlands	retrospective cohort study	2013–2017	60 %	Sepsis	31.10 %	155	66.0(13.6)	SMI	in-hospital mortality	CT
Hwang 2019 [22]	CT scan at the admission	USA	retrospective cohort study	2012–2014	59 %	Trauma	32.00 %	230	≥ 55 years	SMI	in-hospital mortality	CT
Joyce 2020 [23]	Seven days prior to and one day after ICU admission	Australia	retrospective observational study	2018–2019	58.4 %	Critically ill	67.70 %	279	63.7 (16.4)	SMA	30-day survival	CT

Abbreviations: *SMI* skeletal muscle index; *ROS* retrospective observational study; *PCS* prospective cohort study; *NA* not available; *SMA* skeletal muscle area; *TPA* total psoas muscle area; *MCSA* masseter cross-sectional area; *CT* Computed Tomography

### Study quality

None of the studies was a randomized controlled study, and study quality was relatively moderate, ranging from 5 to 8 points NOS (Table S3).

### Mortality

**Fig. 2:**
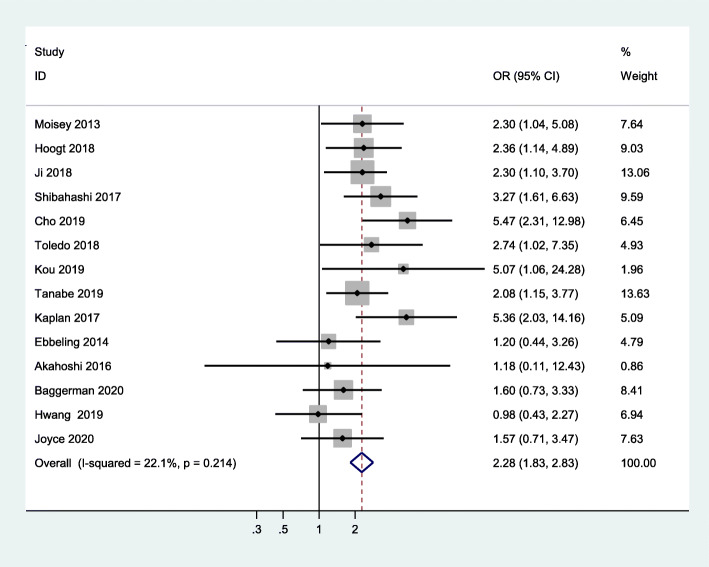
Meta-analysis of the association between sarcopenia and mortality in critically ill patients

**Fig. 3:**
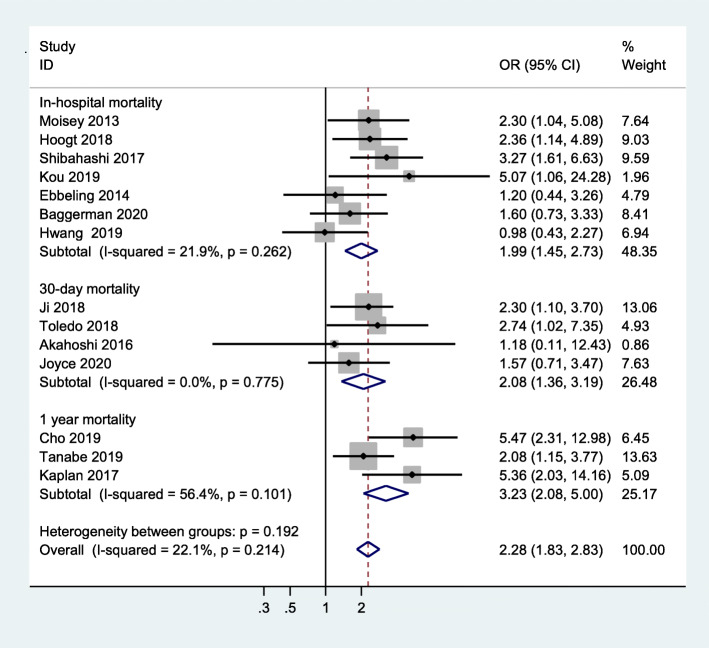
Subgroup meta-analysis of the association between sarcopenia and mortality in critically ill patients by outcome type

All studies that were included used mortality as the primary outcome. Our study showed that critically ill patients with sarcopenia have an increased risk of mortality when compared to those without sarcopenia (OR = 2.28, 95 %CI:1.83–2.83, I^2^ = 22.1 %) (Fig. 2). In addition, pooled data showed a significantly high risk of in-hospital mortality in critically ill patients with sarcopenia, compared to non-sarcopenic patients (OR = 1.99, 95 %CI:1.45–2.73, I^2^ = 21.9 %), 30-day mortality (OR = 2.08, 95 %CI:1.36–3.19, I^2^ = 0.0 %), and 1-year mortality (OR = 3.23, 95 %CI:2.08-5.00,I^2^ = 56.4 %) (shown in Fig. 3). Meanwhile, we examined the minimum sample size required by trial sequential analysis for meta-analysis and found that 1,105 participants were required. There were 3,249 participants included from these 14 studies. In addition, as we can see the Z line has crossed both information size and conventional boundaries, indicating that the association between sarcopenia and all-cause mortality in our analysis was reliable and robust. (Figure S2).

### Subgroup analyses

#### Reasons for ICU admission

**Fig. 4:**
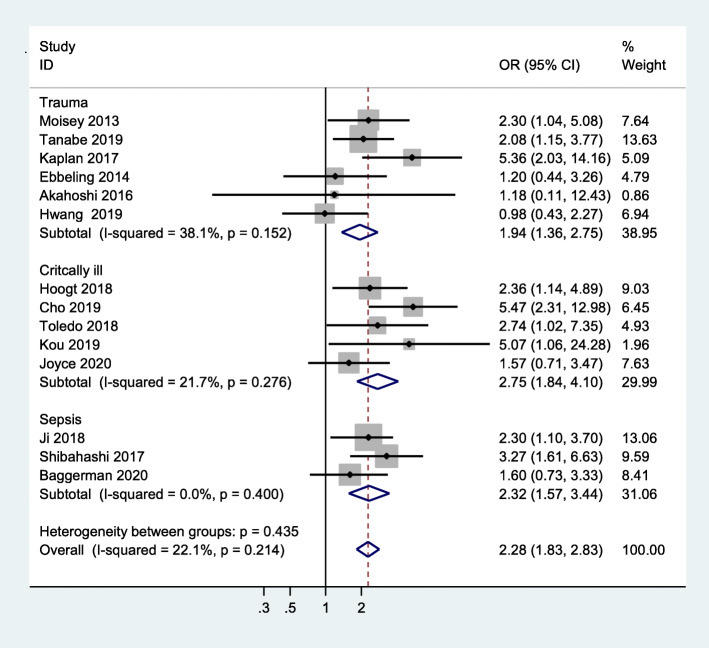
Subgroup meta-analysis of the association between sarcopenia and mortality in critically ill patients by cause of disease

Six studies clearly reported that the reason for admission to ICU was trauma [10, 22, 24, 25, 33, 34], while three studies indicated sepsis as the reason for admission [11, 17, 35]. The patients in the other studies showed complex reasons for ICU department admittance [18–21, 23]. Therefore, we performed a subgroup analysis of the reasons for admission, and found that patients with sarcopenia had an increased mortality risk when compared to patients without sarcopenia among sepsis patients (OR = 2.32, 95 % CI:1.57–3.44; I^2^ = 0.0 %). The results were similar among trauma patients (OR = 1.94, 95 %CI:1.36–2.75, I^2^ = 38.1 %) and patients admitted for other mixed reasons (OR = 2.75, 95 % CI:1.84–4.10, I^2^ = 21.7 %) (Fig. 4).

#### Different definitions of sarcopenia

**Fig. 5:**
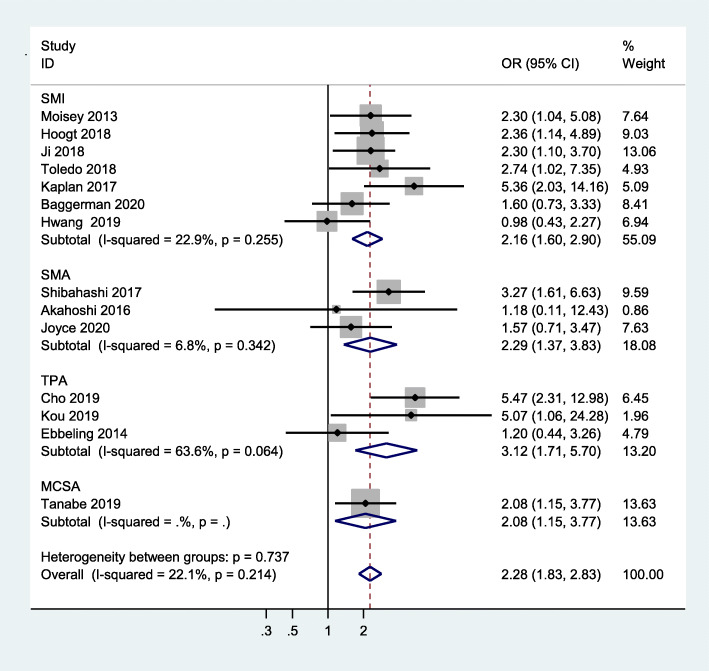
Subgroup meta-analysis of the association between sarcopenia and mortality in critically ill patients by different definition

There are several methods to measure skeletal muscle mass, including total skeletal muscle area or psoas muscle area, as well as masseter cross-sectional area. Detailed information, including muscle measurement and cut-off values, are shown in Table 2. Therefore, we performed a subgroup analysis based on different measures to detect whether there was a difference. Our results showed that critically ill patients with sarcopenia had an increased risk of mortality, compared with non-sarcopenic patients, when using SMI to define sarcopenia (OR = 2.16, 95 %CI:1.60–2.90, I^2^ = 22.9 %). In addition, we found similar results when using TPA (OR = 3.12, 95 % CI: 1.71–5.70, I^2^ = 63.6 %) or SMA (OR = 2.29, 95 %CI:1.37–3.83, I^2^ = 6.8 %) to define sarcopenia. The association between sarcopenia and mortality, based on masseter cross-sectional area, was also significantly different (OR = 2.08, 95 %CI:1.15–3.77) (Fig. 5).

**Table 2:** Computed Tomography Assessment of sarcopenia

Study	Muscles Measured	Level	Cut-off value/Definition
Moisey 2013	Skeletal Muscle Index	L3	Males < 55.4cm^2^/m^2^ Females < 38.9cm^2^/m^2^
Hoogt 2018	Skeletal muscle area index	L3	No definition of sarcopenia
Ji 2018	Skeletal muscle area index	L3	Male < 40.8cm^2^/m^2^ Female < 34.9cm^2^/m^2^
Shibahashi 2017	Skeletal muscle area	L3	Men < 45.2 cm^2^ Women < 39.0 cm^2^
Cho 2019	psoas cross-sectional area	L3	Males < 5.45cm^2^/m^2^ Female < 3.85cm^2^/m^2^
Toledo 2018	Skeletal muscle area index	L3	Male < 55.27 cm2/m2 Female < 40.13 cm2/m2
Kou 2019	Total psoas area	L3	Male < 545mm^2^/m^2^ Female < 385 mm^2^/m^2^
Tanabe 2019	Masseter cross-sectional area	2 cm below the zygomatic arch in the axial plane	Male < 438.6 (100.2) mm^2^ Female < 347.8 (87.5) mm^2^
Kaplan 2017	Skeletal muscle area index	L3	Males < 52.4 cm^2^/m^2^ Female < 38.5cm^2^/m^2^
Ebbeling 2014	Psoas: L4Vertebral Index	L4 inferior body	< 50 percentile of PLVI(≤ 0.83)
Akahoshi 2016	Skeletal muscle area	L3 caudal end	Measured SMA < 80 % estimated SMA
Baggerman 2020	Skeletal muscle area index	L3	Males < 41.6 cm^2^/m^2^ Females < and 32.0 cm^2^/m^2^
Hwang 2019	Skeletal muscle area index	L3	Males < 55.4 cm^2^/m^2^ Females < 38.9 cm^2^/m^2^
Joyce 2020	Skeletal muscle area	L3	Females < 110 cm^2^ Males < 170 cm^2^

#### Subgroup analyses according to region

**Fig. 6:**
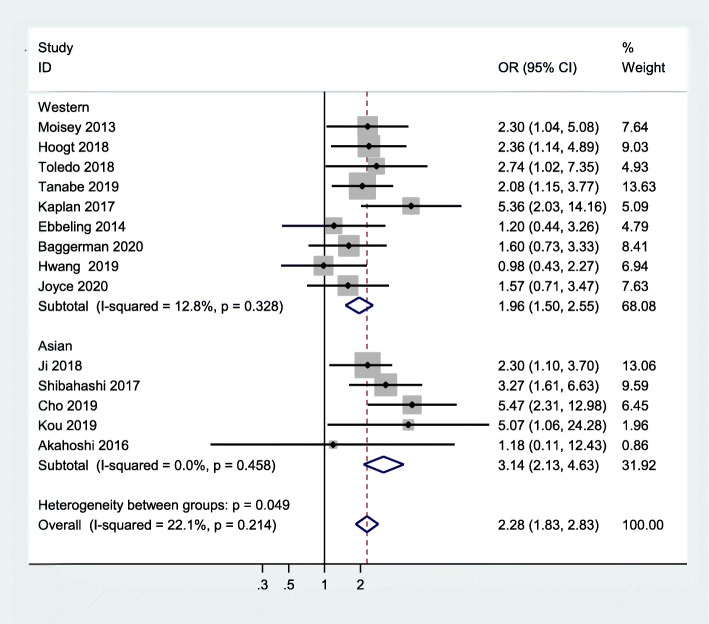
Subgroup meta-analysis of the association between sarcopenia and mortality in critically ill patients according to region

Five studies were conducted in Asian populations [17, 20, 21, 24, 35], and nine studies in Western populations [10, 11, 18, 19, 22, 23, 25, 33, 34]. Therefore, we performed a subgroup analysis based on geographical region. The results showed that critically ill Asian patients with sarcopenia have an increased risk of mortality, compared to critically ill Asian patients without sarcopenia (OR = 3.14, 95 % CI:2.13–4.63; I^2^ = 0.0 %). Similar results were found in Western populations (OR = 1.96, 95 % CI:1.50–2.55;I^2^ = 12.8 %). Figure 6 summarises the results.

#### Subgroup analysis by age

As age is an important confounding factor, we performed a subgroup analysis based on two age groups (more than or equal to 70 years versus less than 70 years). The results showed that the association between mortality and sarcopenia was observed in both of these age groups (OR = 2.33, 95 %CI:1.63–3.33; I^2^ = 0.0 % versus OR = 2.25, 95 %CI:1.70–2.96; I^2^ = 38.5 %, respectively). See Figure S3.

#### Publication bias and sensitivity analysis

The results of Begg’s and Egger’s tests show no significant bias (*P* = 0.956, *P* = 0.785, respectively) (Figure S4). In addition, sensitivity analysis results show that the pooled result did not result in significant change after one study was omitted each time (Figure S5).

#### Overall evidence quality

Our study indicates the quality of evidence was low due to the risk of bias, indirectness, and imprecision (Table S4).

## Discussion

Our study found that critically ill patients with sarcopenia have a 2.28-fold(95 %CI:1.83–2.83) increased risk of mortality compared to patients without sarcopenia, regardless of short– or long-term mortality. To the best of our knowledge, this is the first significant comprehensive study to systematically summarise evidence on the association between sarcopenia and mortality in intensive care units. Our study suggests that intensive care physicians should focus more on screening for sarcopenia and should recommend early and effective preventive programmes, such as resistance training or nutrition treatments, with the goal of reducing patient mortality rates in ICU departments.

The pooled prevalence of sarcopenia was 41 % (95 %CI: 33- 49 %), which is higher than among community-dwelling older adults [36]. In fact, the prevalence of sarcopenia varied among different participants and was also determined by measurements detecting muscle mass with different cut-off points. A recent study about mechanically ventilated critically ill patients found that the prevalence of sarcopenia assessed by SARC-F Questionnaire was 30.2 % [37], which was lower than that in our study. In fact, a systematic review reported that the sensitivity of SARC-F was poor as 0.21 [95 % (CI),0.13–0.31], which leads to underestimate the prevalence of sarcopenia [38]. Other study showed that prevalence of sarcopenia among hospitalized patients who need mechanical ventilation was as high as 60 % [39]. Long-time bedrest and immobilization accelerated skeletal muscle mass loss. Therefore, it is not surprising to observe that the higher prevalence of sarcopenia among critically ill is still needed to be stressed by clinicians.

There were five included studies reported that critically ill patients with sarcopenia did not have a significantly increased risk of mortality than those without sarcopenia. The main reason was that all of these above-mentioned studies did not have enough sample size with wide 95 % CI for effect size. After using meta-analysis by fixed model, sarcopenia was significantly associated with a higher risk of mortality. The main reasons accounting for patient admission to ICU are sepsis or cancer-related infection or trauma [40–42]. Our subgroup results found that when considering the reasons for admission, critically ill patients with sarcopenia have an increased risk of mortality, profoundly confirming that sarcopenia could be a prognostic factor in critical illness. Our findings are in line with previous research, which has found that older adults with sarcopenia are at increased risk of mortality in other settings, such as the community [36], nursing homes [43], or in an oncology setting [44–46]. According to published studies, the main reason explaining the relationship between sarcopenia and mortality is lower muscle mass, which has been confirmed as a strong predictor for an increased risk of death [31, 47]. Apart from these factors, critically ill patients often experience complications from worsening conditions, such as severe inflammation, malnutrition, and multiple organ failure, which make patients ill in a vicious circle through the interaction of sarcopenia [48]. Furthermore, being critically ill with sarcopenia may aggravate the possibility of adverse effects resulting from intensive care treatments, including polypharmacy, bed rest, sedation, instrumentation, and mechanical ventilation. All of these multiple factors will increase the risk of mortality in critically ill patients with sarcopenia.

There are several definitions for sarcopenia in these included studies, based on CT scan and the cut-off for low skeletal muscle mass [16]. Therefore, we performed a subgroup analysis based on methods to measure skeletal muscle mass. By using different measurements, including SMI, SMA, MCSA, and TPA, our subgroup analysis found that critically ill patients with sarcopenia are at increased risk of mortality, compared with non-sarcopenic critically ill patients. There is no consensus on cut-off for CT scans in defining sarcopenia, leading to differing rates of sarcopenia prevalence and problems in standard clinical practice [49]. According to the latest European Working Group on Sarcopenia in Older People (EWGSOP) update, defining sarcopenia should include an assessment of strength, muscle mass, and detecting sarcopenia severity based on physical performance [50]. However, it is difficult for medical staff to detect strength and physical performance in a critical care setting. The definition of sarcopenia based on a CT scan of muscle mass is currently routine application for critically ill patients, particularly trauma patients, but further studies are required to testify to a standard criteria definition of sarcopenia that can be applicable in clinical practice.

Body composition in people of different ethnicities can vary, given the variety between populations [51]. It is obvious that ethnic and environmental factors, such as industrialization, may lead to varying lifestyles and levels of physical activity, which can affect body composition. Using the same cut-off values for different ethnicities can be problematic. Several academic organizations, such as EWGSOP and The Asian Working Group for Sarcopenia (AWGS) [52] have formulated different sarcopenia criteria. These two criteria have widely been used in both Asian and Western countries, but with different cut-off values for low skeletal muscle mass. This meta-analysis included many studies from different counties. Our subgroup analysis shows that people who are critically ill with sarcopenia are at increased risk of mortality in both Asian and Western populations, indicating that sarcopenia’s impact on critically ill patients is not affected by ethnicity.

Obesity was another important and common condition among critically ill patients, with a prevalence rate of 20 % among ICU patients [53]. The “obesity paradox” is a well-known phenomenon in most chronic wasting diseases. A recent study confirmed the “obesity paradox” (proven in e.g. chronic heart failure or dialysis patients) among hospitalized and ICU patients, indicating a J-shaped association between BMI and mortality. This indicates that moderate obesity was a protective factor for critically ill patients, compared to normal or more severe obesity [54]. However, this study did not adjust muscle mass. Critically ill patients with sarcopenia can coexist with obesity, which is called sarcopenic obesity [55]. A previous review revealed that adults with sarcopenic obesity could be at increased risk for all-cause mortality in different settings [56]. Critically ill patients with sarcopenic obesity have been reported to amplify the risk of mortality [57]. The main reason might be that obese individuals have some characteristics of muscle including fiber-type modifications, mitochondrial dysfunction, lower capillary density, which were linked to impaired functionality [58]. Thus, these changes in body composition combined with sarcopenia could aggravate the risk of morality.

### Implications for clinicians, policy, and research

A crucial aspect of our study is to confirm whether ICU care processes could be amended to improve clinical outcomes in patients with sarcopenia. Recent studies have determined processes that may exert an important impact on sarcopenic patients, include resistance training programmes [59], nutritional support, and intensity of rehabilitation [60]. While research is being conducted on how to reduce patient mortality rates in intensive care units, realising sarcopenia as a risk factor in mortality is also significant, and may help in more effective care planning. To date, many conventional scoring systems, including Acute Physiology and Chronic Health Evaluation (APACHE), sequential organ failure assessment (SOFA), and systemic inflammatory response syndrome (SIRS) have been used to assess critically ill patients [6, 61]. However, published studies have shown their performance in predicting mortality is modest and cannot satisfy intensive care physicians [62]. Our study supports the value of sarcopenia screening upon ICU admission. Whether measuring muscle mass using CT can improve mortality prediction by adding to conventional scoring systems is worthy of study, given the convenience and simplicity of CT scans.

Our study has a number of strengths. First, to the best of our knowledge, this is the first meta-analysis to quantify the relationship between sarcopenia and mortality in critically ill patients using comprehensive methods and low heterogeneity. Second, we assessed our study using GRADE and recommend using the level of evidence to help medical staff guide clinical work. Our study may also have some limitations. First, the studies that were included in our meta-analysis were all observational studies. They might contain biases, such as selection and confirmation, and cannot determine causation. Second, five studies reported that sarcopenia detection occurred within several days after ICU department admission, meaning that sarcopenia could be a consequence of disease severity. Therefore, this meta-analysis could intensify sarcopenia’s impact on mortality. Third, there are several different measurements of muscle mass with various SMI cut-offs to determine sarcopenia, resulting in a differing prevalence of sarcopenia and which could eventually lead to different results. Various cut-off of SMI, without a universally agreed consensus on cut-off values for low skeletal muscle, can generate a problem that influences the treatment of patients for sarcopenia. According to the study of Yoowannakul, cut-off values for low skeletal muscle should be adjusted according to gender, normative ethnicity, and age values [63]. When we conducted a subgroup analysis based on muscle measurement, we found the results were similar across various sarcopenia measures. However, unanswered questions remain as to which is the most appropriate measure in an ICU setting. Fourth, most studies performed multivariate analysis to investigate the relationship between sarcopenia and mortality in critically ill patients, yet not all studies adjusted the same confounders, which may have resulted in an underestimation or overestimation of our results. In addition, a number of important confounding factors, such as chronic obstructive pulmonary disease (COPD), obesity, and cardiac failure, which would have influenced sarcopenia’s impact on mortality, were not reported in the original studies. Therefore, we could not conduct a subgroup analysis based on these parameters. Fifth, the studies that were included were from different countries, with different hospital systems and a variety of medical technologies and healthcare settings, which could have influenced the outcomes. Sixth, there are different types of outcomes, including in-hospital mortality, 30-day mortality, and 1-year mortality, which might have exerted an adverse impact on aggregation. Seventh, two studies used the effect measure with HR not OR; when we pooled the total effect size, we considered HR as OR; therefore, it might overestimate or underestimate the results.

## Conclusions

Our study found that critically ill patients with sarcopenia have an increased 2.28-fold(95 %CI:1.83–2.83) risk of mortality compared to those without sarcopenia. Timely routine assessment for sarcopenia upon ICU admission may provide an important prognostic factor in patient survival. Offering corresponding interventions may help medical staff achieve good patient outcomes. Furthermore, this study also suggests the importance of initiating effective intervention programmes, such as resistance training and appropriate nutritional treatment, which may reduce the risk of mortality.

## Supplementary Information

**Additional file 1: Table S1**: PRISMA checklist

**Additional file 2.** Search Strategy

**Additional file 3: Table S2**: Results of all the studies by using Multivariate Logistic Regression for adjusting covariates. **Table S3**: Result of the Newcastle-Ottawa scale quality assessment. **Table S4**: Overall evidence quality. **Figure S1**: The flow diagram of studies selection. **Figure S2**: The results of trial sequential analysis on mortality. **Figure S3**: Subgroup meta-analysis of the association between sarcopenia and mortality in critically ill patients between different age. **Figure S4**: Begg's and Egger's test for publication bias. **Figure S5**: Sensitivity analysis of all studies.

## Data Availability

All the data can obtain from the PubMed database(https://pubmed.ncbi.nlm.nih.gov).

## Abbreviations

CT: Computed tomography
Mesh: Medical subject headings
NOS: Newcastle Ottawa Scale
OR: Odds ratio
HR: Hazard ratio
SMI: Skeletal muscle index
SMA: Skeletal muscle area
TPA: Total psoas muscle area
MCSA: Masseter cross-sectional area
