# Supplementary material for: Sarcopenia as a predictor of mortality among the critically ill in an intensive care unit: a systematic review and meta-analysis
Source: BMC Geriatr. 2021 Jun 2;21:339. doi: 10.1186/s12877-021-02276-w (PMC8173733; doi:10.1186/s12877-021-02276-w)
Supplement: Supplementary file 2 — Additional file 2. Search Strategy [file 12877_2021_2276_MOESM2_ESM.doc]

Search strategy by Pubmed

#3 Add Search (sarcopeni*) AND ((((((intensive care unit) OR intensive care) OR "Critical Care"[Mesh]) OR critically ill) OR critical illness) OR "Critical Illness"[Mesh]) 286 04:47:31

#2 Add Search sarcopeni*

#1 Add Search (((((intensive care unit) OR intensive care) OR "Critical Care"[Mesh]) OR critically ill) OR critical illness) OR "Critical Illness"[Mesh]
