# Supplementary material for: Sarcopenia as a predictor of mortality among the critically ill in an intensive care unit: a systematic review and meta-analysis
Source: BMC Geriatr. 2021 Jun 2;21:339. doi: 10.1186/s12877-021-02276-w (PMC8173733; doi:10.1186/s12877-021-02276-w)
Supplement: Supplementary file 3 — Additional file 3: Table S2: Results of all the studies by using Multivariate Logistic Regression for adjusting covariates. Table S3: Result of the Newcastle-Ottawa scale quality assessment. Table S4: Overall evidence quality. Figure S1: The flow diagram of studies selection. Figure S2: The results of trial sequential analysis on mortality. Figure S3: Subgroup meta-analysis of the association between sarcopenia and mortality in critically ill patients between different age. Figure S4: Begg's and Egger's test for publication bias. Figure S5: Sensitivity analysis of all studies. [file 12877_2021_2276_MOESM3_ESM.doc]

Table S2: Results of all the studies by using Multivariate Logistic Regression for adjusting covariates.

| Study | Adjustment |
| --- | --- |
| Moisey 2013 | Age,sex, Injury Severity Scale score |
| Ebbeling 2014 | Age, Comorbidities, Abbreviated Injury Scale head |
| Kaplan 2016 | Age, sex, and comorbidities |
| Akahoshi 2016 | Age, sex |
| Shibahashi 2017 | APACHE II score, sex |
| Hoogt 2018 | NA |
| Ji 2018 | Age, use of vasopressor, mixed organism, SOFA |
| Toledo 2018 | NA |
| Cho 2019 | NA |
| Kou 2019 | NA |
| Tanabe 2019 | Age, comorbidity,complications,mechanism of injury, Injury Severity Score, Abbreviated Injury Scale scores |
| Hwang 2019 | NA |
| Baggerman 2020 | NA |
| Joyce 2020 | NA |

*Acute Physiology and Chronic Health Evaluation II score

Sequential Organ Failure Assessment score

NA：not available

Table S3: Result of the Newcastle-Ottawa scale quality assessment

| Newcastle-Ottawa scale | Selection(1) |  |  |  | Comparability(2) | Outcome(3) |  |  | Total |
| --- | --- | --- | --- | --- | --- | --- | --- | --- | --- |
|  | Representativeness  of the exposed cohort | Selection of the non-exposed cohort | Ascertainment of exposure | Demonstration that outcome of interest was not present at start of study | Comparability of cohorts on the basis of the design or analysis | Assessment of outcome | Was follow-up long enough for outcome to occur | Adequacy of follow up of cohorts |  |
| Moisey 2013 | 1 | 1 | 1 | 1 | 2 | 1 | 0 | 1 | 8 |
| Hoogt 2018 | 1 | 1 | 1 | 1 | 1 | 1 | 0 | 1 | 7 |
| Ji 2018 | 1 | 1 | 1 | 1 | 1 | 1 | 0 | 1 | 6 |
| Shibahashi 2017 | 1 | 1 | 1 | 1 | 0 | 1 | 0 | 1 | 6 |
| Cho 2019 | 1 | 1 | 1 | 1 | 1 | 1 | 1 | 1 | 8 |
| Toledo 2018 | 1 | 1 | 1 | 1 | 1 | 1 | 0 | 1 | 7 |
| Kou 2019 | 1 | 1 | 1 | 1 | 1 | 1 | 0 | 1 | 7 |
| Tanabe 2019 | 1 | 1 | 1 | 1 | 1 | 1 | 1 | 1 | 8 |
| Kaplan 2016 | 1 | 1 | 1 | 1 | 1 | 1 | 1 | 1 | 8 |
| Ebbeling 2014 | 1 | 1 | 0 | 1 | 1 | 1 | 0 | 1 | 6 |
| Akahoshi 2016 | 0 | 1 | 1 | 1 | 1 | 0 | 0 | 1 | 5 |
| Baggerman 2020 | 1 | 1 | 1 | 1 | 2 | 1 | 0 | 1 | 8 |
| Hwang 2019 | 1 | 1 | 1 | 1 | 1 | 1 | 0 | 1 | 7 |
| Joyce 2020 | 1 | 1 | 1 | 1 | 1 | 1 | 0 | 1 | 7 |

TableS4

Overall evidence quality

| **Quality assessment** | | | | | | | **Number of studies of patients** | | **Effect** | | **Quality** | **Importance** |
| --- | --- | --- | --- | --- | --- | --- | --- | --- | --- | --- | --- | --- |
|
| **Number of studies** | **Design** | **Risk of bias** | **Inconsistency** | **Indirectness** | **Imprecision** | **Other considerations** | **Sarcopenia** | **Non-sarcoepnia** | **Relative (95% CI)** | **Absolute** |
| **Mortality (follow-up median 6 months)** | | | | | | | | | | | | |
| 14 | observational studies | serious^1^ | no serious inconsistency^1^ | no serious indirectness^1^ | no serious imprecision | strong association^1^ | - | - | OR 2.28 (1.83 to 2.83) | - | ÅÅOO LOW | CRITICAL |

Supplemental Figure S2: The results of trial sequential analysis on mortality


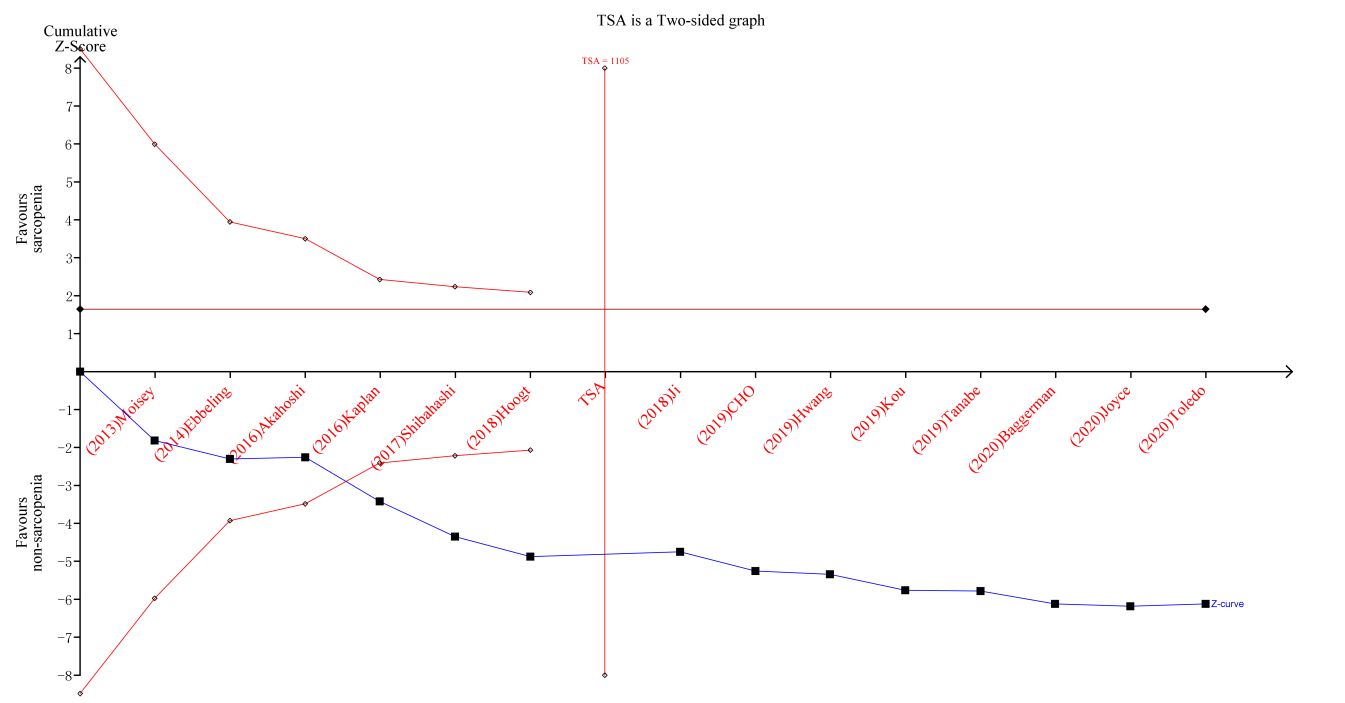


Figure S3**:** Subgroup meta-analysis of the association between sarcopenia and mortality in critically ill patients between different age

Supplement Figure S4: Begg’s and Egger’s test for publication bias.

Supplement Figure S5**.** Sensitivity analysis of all studies.
